# Potential of different cells-derived exosomal microRNA cargos for treating spinal cord injury

**DOI:** 10.1016/j.jot.2021.09.008

**Published:** 2021-10-25

**Authors:** Dayu Pan, Weixiao Liu, Shibo Zhu, Baoyou Fan, Nanxi Yu, Guangzhi Ning, Shiqing Feng

**Affiliations:** a Department of Orthopedics, Tianjin Medical University General Hospital, Heping District, Tianjin, 300052, PR China; b Tianjin Neurological Institute, Key Laboratory of Post-Neuro Injury Neuro-repair and Regeneration in Central Nervous System, Ministry of Education and Tianjin City, Heping District, Tianjin, 300052, PR China; c Department of Endocrinology, Endocrinology Research Center, Xiangya Hospital of Central South University, Changsha, Hunan, China

**Keywords:** Spinal cord injury, MicroRNA, Exosome

## Abstract

Spinal cord injury (SCI) is a disastrous situation that affects many patients worldwide. A profound understanding of the pathology and etiology of SCI is of great importance in inspiring new therapeutic concepts and treatment. In recent years, exosomes, which are complex lipid membrane structures secreted nearly by all kinds of plants and animal cells, can transport their valuable cargoes (e.g., proteins, lipids, RNAs) to the targeted cells and exert their communication and regulation functions, which open up a new field of treatment of SCI. Notably, the exosome's advantage is transporting the carried material to the target cells across the blood–brain barrier and exerting regulatory functions. Among the cargoes of exosomes, microRNAs, through the modulation of their mRNA targets, emerges with great potentiality in the pathological process, diagnosis and treatment of SCI. In this review, we discuss the role of miRNAs transported by different cell-derived exosomes in SCI that are poised to enhance SCI-specific therapeutic capabilities of exosomes.

## 1 Introduction

**Fig. 1:**
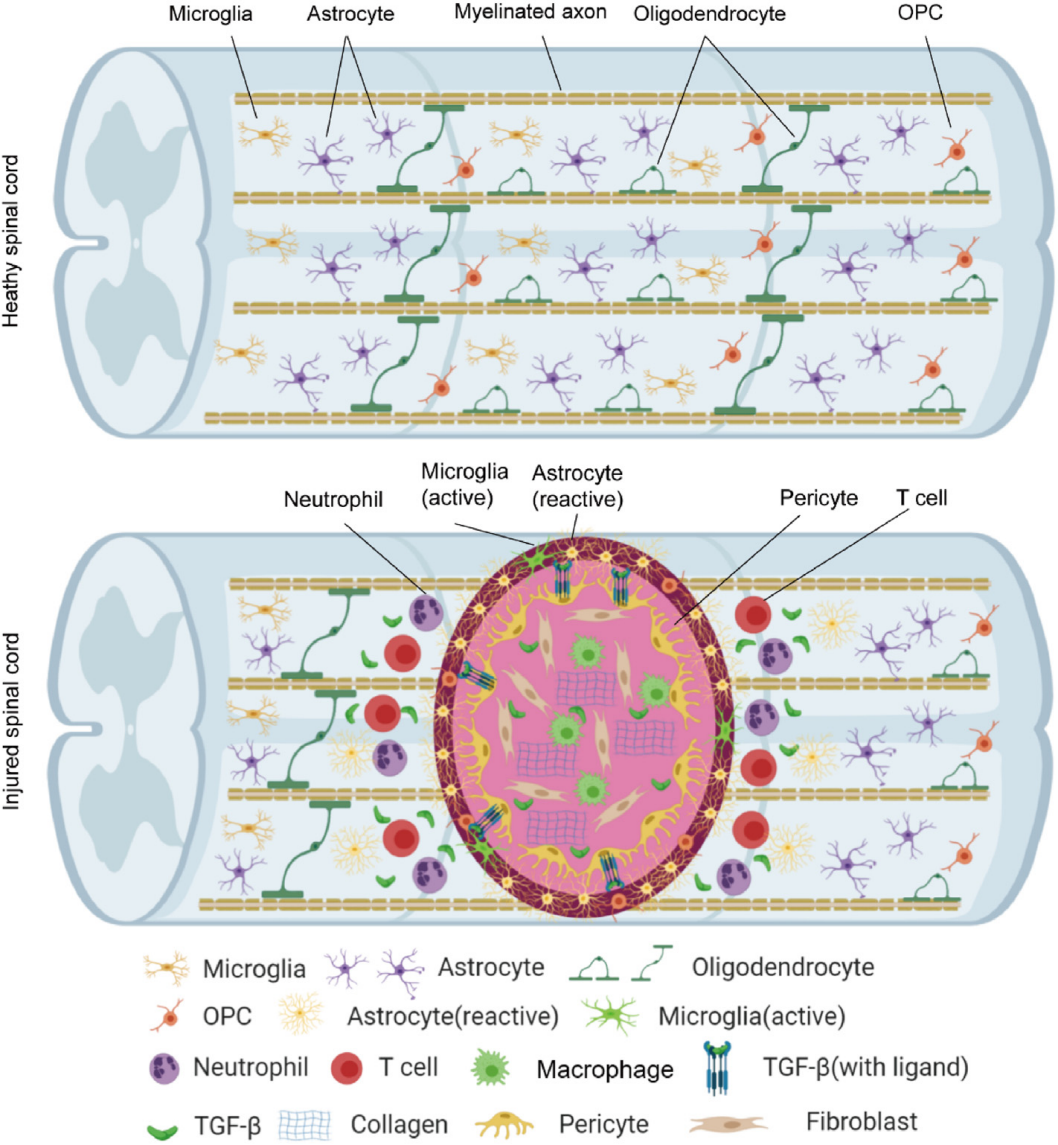
Schematic diagram of SCI. The upper diagram shows the composition of the intact spinal cord, and the lower diagram shows the synthesis of the spinal cord at various stages after SCI.

**Fig. 2:**
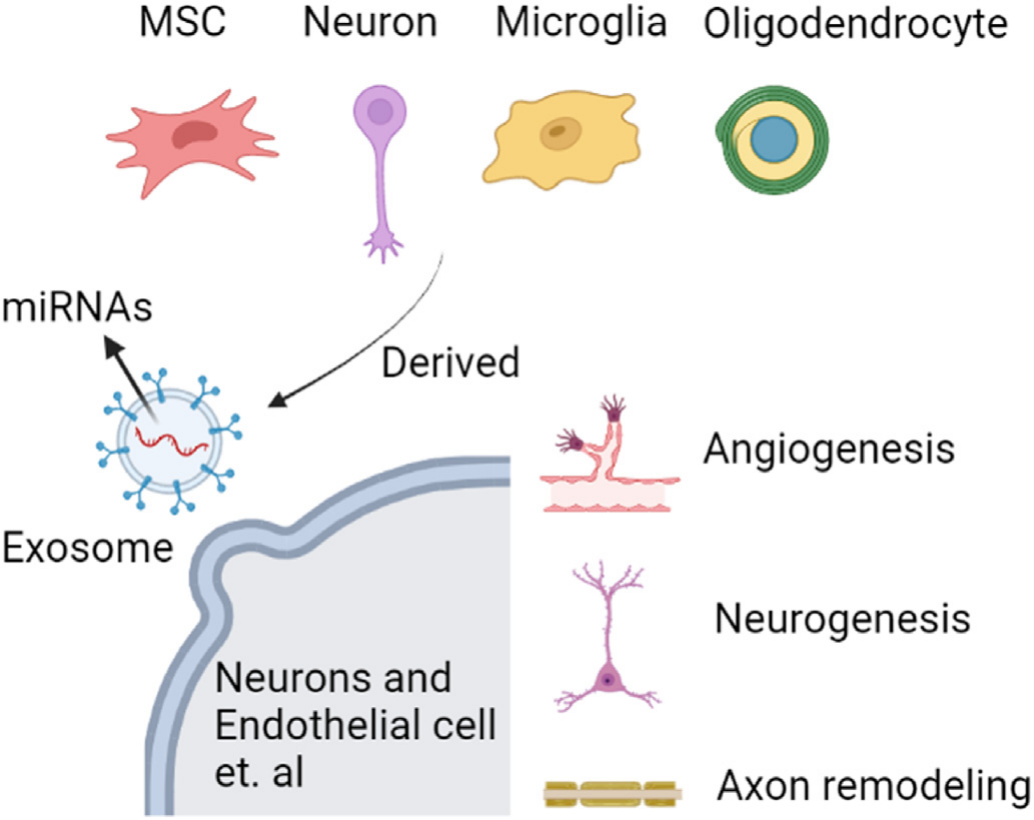
The functions of different cell-derived exosomes.

SCI is a devastating neurological disorder that causes severe physical and psychological injury to the patient and brings a substantial economic burden to society [1,2]. It is a common injury with complex and disastrous clinical and pathological processes separated into immediate mechanical primary damage and secondary cascade damage [3,4]. According to current knowledge, multiple factors are involved in secondary damage: blood–brain barrier dysfunction, local inflammation, glia or fibrotic scar formation, neuronal death, demyelination and disruption of neural pathways (Fig. 1) [2,5]. After the primary injury, the infiltration of activated microglia and peripheral immune cells triggers a robust neuroinflammatory response. Monocytes infiltrate and occupy the center of the injury site to remove tissue debris. T and B lymphocytes also infiltrate the spinal cord in the subacute phase and produce pro-inflammatory cytokines, chemokines, autoantibodies, reactive oxygen species and nitrogen substances. The loss of oligodendrocytes in the acute and subacute phases of SCI leads to axon demyelination, followed by spontaneous remyelination in the subacute and chronic phases. Astrocytes and Oligodendrocyte progenitor cells (OPCs) proliferate in the spinal cord parenchyma and migrate to the injured site, helping to form glial scars. Pericytes, fibroblasts, and released collagen and fibronectin together form fibrotic scars (Fig. 1). In addition, mesenchymal stem cells (MSCs) secrete a number of neurotrophic factors, such as brain-derived growth factor (BDNF), glial-derived growth factor (GDNF), nerve growth factor (NGF), Neurotensin-1 (NT-1), Neurotensin-3 (NT-3), Ciliary Neurotrophic Factor (CNTF), and basic fibroblast growth factor (bFGF) [[6], [7], [8], [9], [10], [11]]. Moreover, MSCs can not only prevent nerve degeneration and apoptosis, but also support neurogenesis, axonal growth, re-myelination, and cell metabolism [[12], [13], [14], [15], [16], [17], [18], [19]] (Fig. 2).

As for SCI treatment, current therapies involve Methylprednisolone (MP), which is a drug approved by both European medical institutions and the Food and Drug Administration (FDA) for 48 ​h in high doses in the acute phase [20]. MP is a corticosteroid that make an inhibition of lipid peroxidation as a free radical scavenger. It also inhibits the inflammatory activities, protects the blood-spinal cord barrier, and promotes blood flow to the spinal cord. Whereas the side effects like urinary tract infection, respiratory infection as well as wound surface infection limit its’ broad utilization [20,21]. According to the pathologic process of central nervous system damage, the current therapeutic theory is composed of neuroprotective treatment and neurorestorative treatment. The neuroprotective treatment focuses on the reduction/prevention of secondary damage nerve cell death and damage size. And the neurorestorative treatment aims to enhance neurological recovery by neurovascular remodeling, involving angiogenesis, neurogenesis, oligodendrogenesis and the outgrowing of dendrite/axon [22].

Exosomes released by oligodendrocytes and the internalization of neurons play an important role in increasing neuronal activity under situations of cellular stress (hypoxia and glucose deficiency). Microglia can also secrete exosomes that contain cytokine interleukin-1β (IL-1β) proprotein. Furthermore, when exosomes are exposed to high levels of extracellular ATP-releasing by astrocytes or damaged tissue, the purinergic (P2X7) receptors of micro vesicles are activated, resulting in caspase-1 mediated leukocyte-mediated division of interleukin-1β proprotein and secrete the mature interleukin-1β from the vesicles [27]. This process may be the promoter of the primary inflammation during SCI. Besides, recent studies have indicated that the effect of stem cells on stroke and traumatic brain damage relied on the production and release of exosomes, which provides the basis for exosome treatment [5,28,29]. Importantly, exosomes could carry proteins, lipids, and RNAs to transport messages among various cell types that affect the normal physiological condition and pathological state [23]. And exosomes are capable of transporting cargoes across blood brain barrier (BBB) and blood-spinal cord barrier (BSCB) to reach distant organs without significant degradation [30]. Moreover, exosomes participate in various biological processes in SCI, including synaptic plasticity, regulating the myelin membrane biogenesis, and local delivery of proteins or nucleic acids to increasingly polarized structures [24,25]. In addition, due to phospholipid bilayers, the inside cargoes are well protected to be delivered to specific target cells [26].

Furthermore, exosomes have emerged as alternatives to cell-based therapies owing to their potential for improved safety and therapeutic efficacy across diverse regenerative applications [10]. Recent studies have demonstrated that exosomes can serve as both positive and negative cues for axonal regeneration [[31], [32], [33], [34], [35]]. Neurite outgrowth inhibitor-A (Nogo-A), a myelin-associated inhibitor (MAI) protein, could be transported by exosomes to inhibit axon growth by binding to Nogo receptor 1 (NgR1) on neurons [35]. By contrast, endogenous exosomes releasing induced by an agonist for retinoic acid receptor β (RARβ) could improve axonal regeneration in a rat SCI model [[32], [33], [34]]. Following treatment, neuronal exosomes transferred phosphatase and tensin homolog (PTEN) into astrocytes, which lead to a dual benefit: extrusion of PTEN improved the intrinsic growth capacity by removing inhibition of the PI3K/Akt/mTOR pathway, whereas transfer of PTEN into astrocytes created a more permissive environment for axon growth by reducing the proliferation of astrocytes and led to glial scar formation [32]. However, the pathophysiological roles of exosomes in the CNS remain largely unknown, hampering rational design of novel exosome-based treatment approaches.

MicroRNA (miRNA) is a small RNA molecule without coding (including approximately 22 nucleotides) discovered among plants, animals, and viruses. They can silence RNA by cleavage of the mRNA strand into two segments. The expression of post-transcriptional genes can be regulated by shortening the poly(A) tail to destabilize the mRNA and reduce the efficient conversion of mRNA to protein [36,37]. There are more than 70% miRNAs expressed in the central nervous system (CNS) and been preserved in species as isochronous molecules [38]. Among the exosomal nucleic acid and silence post-transcriptional mRNA expression, exosomal miRNAs are the most critical functional substances, which are involved in cell proliferation and differentiation, immunomodulation and angiogenesis [30,39,40]. After being sorted in exosomes, the mature miRNAs can be transported to recipient cells to influence the protein network and RNA production of the recipient cells by regulating gene expression and key homeostatic processes. In the middle of the progress of the SCI, many miRNAs are significant regulators. In brief, nerves could be controlled by inhibiting translating multiple mRNA targets and/or the multiple miRNA's effects (e.g., proliferation, migration and differentiation of neural stem cells (NSC) and its progenitor cells [[41], [42], [43]]. The decreasing effect of Dicer or Drosha (or its co-factor DGSR8) confirms the role of miRNAs in neurodevelopment, which are important sections of miRNA biogenesis [[44], [45], [46]].

## 2 Exosomal miRNAs and SCI

As mentioned above, exosomes serve as a third type of intercellular communication vector and own an irreplaceable position in the functional integrity of multicellular organisms. Protected by the phospholipid bilayer, these cargos not only can be accurately transported to the target tissue but also effectively avoid the hydrolysis of various enzymes in the extracellular matrix.

In 2007, Hai Valadi et al. claimed a novel intercellular communication mechanism that exosomes can deliver mRNA and small RNAs between cells, such as miRNAs. And they proposed that this kind of RNA is called “exosomal shuttle RNA” (esRNA) [47]. This genetic communication between cells may potentially occur at a distance by exosomes through the systemic circulation. And the ability to modify recipient cell protein production and gene expression by specific mRNA or miRNA delivered by exosomes make them to be ideal candidate for gene therapy [48].

Exosome-mediated circulating miRNA is a new way of intercellular [49] gene transfer among cells and a biomarker for many diseases [[50], [51], [52]]. The exosome shuttle miRNA is fused with multivesicular bodies (MVBs) and secreted by the endosomal membrane compartment, which can be transported long distances in body fluids [[53], [54], [55]].

Down-regulation of miR-291–3p, −183, −92, −200b, and −200c through the neurogenesis and neural tube (NT) growth in mouse, and inner human central nervous system developmental models, while miR-9, -124a, −7, −125a and −125b are up-regulated [[56], [57], [58]]. MiR-124, -125b, −137 and −9 enhance neuronal differentiation. However, to the contrary, miR-183 and miR-8/−200 anti-apoptotic family is divided into anti-apoptotic, neural progenitor maintenance, and proliferative molecules [59,60].

Except for their physiological function, miRNAs act on the pathogenesis processes of SCI. For example, miR-21 has been detected that determining the shift from hypertrophy to hyperplasia during the process of astrogliosis [3]. By using viral miR-133b infection adult mouse spinal cord model, Thomas Theis, etc. observed reduction of RhoA, xylosyltransferase 1 (*Xylt1*), ephrin receptor A7 (*Epha7*), and purinergic receptor P2X ligand-gated ion channel 4 (*P2RX4*), that has been determined as a negative factor in neurite outgrowth [61]. In addition, mir-494, which was discovered and proved by Huaguang Zhu et al., has the ability to inhibit apoptotic cells, reduce lesion size and improve functional recovery [62]. Furthermore, up-regulation of miR-126 enhances angiogenesis and inhibits leukocyte overflow in the damaged spinal cord [63]. Whereas, due to drug challenges like degradation in the blood and poor target delivery of system-delivered miRNA mimics, and clinical difficulties related to local transfer, the prime research has barely succeeded in applying these methods to clinical practice [64].

## 3 MSCs derived exosomal miRNAs and SCI

Like general exosomes, MSC-derived exosomes carry complex cargo, including proteins, nucleic acids and lipids [[65], [66], [67]]. The miRNAs encapsulated in MSC exosomes mainly exist in the form of their precursors [68]. MSCs have been used to treat central nervous system damage. It has been demonstrated that MSCs generalized axons promotes neurogenesis and angiogenesis, decrease neuroinflammation, decrease model separation and spatial learning disorders, and enhance recovery of function of animal brain injury models [[69], [70], [71]] (Fig. 2). Emerging evidence shows that the efficacy of MSC treatment is mainly derived from releasing nutrient factors through paracrine actions to reduce inflammation, support nerves, and promote regeneration of damaged tissue instead of differentiating and replacing the lost cells in the injury site [72,73]. Extensive studies have indicated that exosomes from MSCs carrying miRNAs have efficient repair effects on SCI [74,75]. Exosomal miRNAs currently studied in SCI mainly include miRNA-486, miRNA-21, miRNA-133b, and miRNA-126 (Table 1).

**Table 1:** Different miRNAs from different cells-derived exosomes and related targets, functions and study models involved in SCI.

Cell (s)	MiRNA (s)	Target (s)/Mechanism (s)	Function	Study model	Ref
**MSC**	miR-486	NeuroD6	Reduce neurons protection	Mice	[76]
	miR-126	RhoA	Promote axonal regeneration Alleviate histopathological damage	Rat	[80]
	miR-29b	NF, GAP43			
	miR-133b	NF, GAP43 CREB and STAT3 RhoA		Rat/Mice/Zebrafish	[82,94,95]
	miR-21	FasL PDCD4 PTEN	Reduce neurons apoptosis Promote functional recovery	Rat/Human	[96]
	miR-19b	PTEN		Human	[78]
	miR-216a-5p	TLR4/NF-κB PI3K/AKT	Promote microglia polarization	Mice	[92]
	miR125-a	IRF5	Promote M2 macropage polarization	Rat	[89]
	miR-124–3p	Ern1		*in vitro*	[90]
	miR199a-3p /145–5p	NGF/TrkA	Promote locomotor function	Rat	[91]
**Neuron**	miR-21	MEF2C	Enhance potassium channel behaviors and expression of nerve important transcription factors	Rat/Human	[[97], [98], [99]]
	miR-146	TLR/NF-κB TRAF5/IRAK1	Promote the repair of SCI and reduce inflammatory responses	Rat	[100]
	miR-7a/b	Zdhhc9/Prkcb/Wipf2/Pfn2	Neurite Outgrowth Reduce apoptosis	Rat	[101,102]
**Microglia**	miR-124–3p	Rela/ApoE	Promote M2 polarization Alleviating neurodegeneration Improve cognitive outcome	Mice	[103]
**Oligoden drocyte**	miR-9 and miR-19a	DCX	Initiate neuronal precursor cell differentiation and allow mature neurons to be polarized	*in vitro*	[53]

Knockdown of miRNA 486 *in vitro* and *in vivo* by small interfering RNAs can effectively improve motor functional recovery and neuroprotection in mice after SCI by inducing the expression of NeuroD6 [76].

MiRNA-21 is one of the most common and most studied miRNAs secreted by MSCs derived exosomes for SCI treatment. Xu et al. [77,78] reported that miRNA-21 regulates apoptosis and differentiation of neurons in patients with SCI by targeting the expression of PTEN or tumor suppressor gene programmed cell death 4 (PDCD4). Interestingly, the protective effect of MSCs derived exosomes could be weakened to reduce the secretion of miRNA-21 by insulin resistance in obese rat [79].

MiRNA-126 was found highly expressed after SCI, while reduced inflammation, increased angiogenesis and improved functional recovery were observed when increasing the level of miRNA-126 by using agomir-126. This process is concurrent with downregulation of expression of Sprouty Related EVH1 Domain Containing 1 (*SPRED1*), Phosphoinositide-3-Kinase Regulatory Subunit 2 (*PIK3R2*) and Vascular Cell Adhesion Molecule 1 (*VCAM1*) target genes [80].

MiRNA-133b plays an important role in neuronal differentiation, growth, and apoptosis [[81], [82], [83]]. Downregulating miRNA-133b by using morpholino antisense oligonucleotides is not conducive to the recovery of motor function and reduces neuronal axonal regeneration after SCI [84]. Li et al. showed increased neurons survival and improved motor function were observed after systemic injection of miRNA-133b exosomes, which were partially due to the ERK1/2, STAT3, CREB and RhoA signaling activation [85]. In addition, MSCs derived exosomal miRNA-133b significantly promote the expression of neurofilament (NF), growth associated protein 43 (GAP-43), glial fibrillary acidic protein (GFAP), and myelin basic protein (MBP) then induce axonal regeneration and promote functional recovery in SCI animals [86]. MSCs-derived exosomes could deliver miR-133b to enhance neurite growth and promote neural plasticity and functional recovery [87,88].

Other miRNAs like miR-125a derived from bone marrow mesenchymal stem cells (BMMSC) exerts neuroprotective effects by targeting and negatively regulating Interferon Regulatory Factor 5 (*IRF5*) expression in SCI rats [89]. MiR-124–3p derived from BMMSCs attenuated nerve injury induced by regulating endoplasmic reticulum to nucleus signaling 1 (*Ern1*) and M2 macrophage polarization [90]. Yang W et al. found that umbilical mesenchymal stem cell-derived exosomal miR-199a-3p/145–5p facilitate spinal cord functional recovery through the mediated NGF/TrkA signaling pathway in rats [91]. Weihua C et al. suggested that miR-216a-5p from MSCs derived exosomes is involved in the modulation of microglial polarization [92].

Therefore, MSC-derived exosomes containing genetic materials (such as miRNA-486, miRNA-21, miRNA-133b, and miRNA-126 et al.) can be used as a cell-free treatment strategy [93], which have great potential to promote functional recovery, and their contents can be used as biomarkers of SCI.

## 4 Neurons derived exosomal miRNAs and SCI

To explore the miRNA's effect in exosomes related to SCI, Emily B and his colleagues purified RNA from mouse brains of traumatic brain injury (TBI) and control groups, then sequenced the miRNA [54,97,98]. They indicated that the miR-212 releasing was significantly reduced with elevating the simultaneous of miR-21 in neurons, indicating miR-21 expressed as potential extracellular vesicle cargos out of neurons. Nevertheless, miR-21, miR-146, miR-7a, and miR-7b all enhanced dramatically after injury [98]. Interestingly, miR-21 mimics treatment enhanced neuroprotective effect in SCI model and overexpression of miR-21 target PTEN [48] reduced the neurotoxicity of the TBI model. Many studies suggested miR-21 plays an important role in neuroprotection and regeneration in stroke [104] and axotomy [105] models. Otherwise, miR-21 is vital in glial cell activities after SCI by decreasing astrocyte hypertrophy and glial scar formation [106]. Moreover, miR-21 targets microglia FasL to reduce microglia-mediated Neuronal death in the stroke model [107]. Although miR-21 expression has potential advantages in neuronal damage, it remains defective. Such as in the human immunodeficiency virus infection (HIV), which is a related neurocognitive disorder, elevated miR-21 leads to enhanced potassium channel behaviors and expression of nerve important transcription factors MEF2C, causing neurological disorders [108]. Recently, Weihua C and his colleagues also found exosomal miR-124–3p derived from neuron can suppress the activation of M1 microglia and A1 astrocytes by attenuating the activity of myosin heavy chain 9 (MYH9) to promote recovery after SCI through PI3K/AKT/NF-κB signaling cascade in mice [109]. In summary, miRNAs, especially miR-21 secreted by neurons might be a great approach to treating the SCI [110].

## 5 Microglia derived exosomal miRNAs and SCI

Shan Huang and his colleagues found that miR-124–3p expression was elevated in exosomes from microglia at 3, 7,14, 21-, and 28-days post TBI. And microglia exosomal miR-124–3p could reduce neurodegeneration and improve cognitive outcome by targeting Rela/ApoE signaling pathway. It is revealed that miR-124–3p can switch cell polarization from the M1 to the M2 phenotype in various subsets of monocyte cells and microglia [111]. Down-regulation of its expression level is an indicator of neuroinflammation in various diseases, such as experimental autoimmune encephalomyelitis [112] intracerebral hemorrhage [93]. At present, researchers confirmed that miR-124–3p benefited anti-inflammatory M2 polarization in microglia and exerted an anti-inflammatory effect on injured neurons via their transfer by microglial exosomes. Thus, these findings suggest that the increased miR-124–3p in microglial exosomes exerts a protective effect in injured brain after TBI [111]. Guofeng C et al. suggested that exosomes derived from M2 microglia alleviates ischemia-reperfusion brain injury through transporting exosomal miRNA-137 targeting Notch1, which indicate a potential therapeutic target for SCI treatment.

## 6 Oligodendrocyte derived exosomal miRNAs and SCI

In the central nervous system, the significant function of oligodendrocytes is providing support and insulation to axons, equivalent to the function of Schwann cells in the peripheral nervous system [113]. Oligodendrocytes includes multivesicular bodies (MVBs) at the axon perimeter, express exosomes involving proteolipid proteins (PLP), myelin proteins, and anti-oxidative stress-related proteins [113,114]. Besides, oligodendroglial precursor cell line Oli-neu can secret exosomes, carrying miR-9 and miR-19a, to decrease neuronal DCX expression. Both miRNAs were predicted to combine with Doublecortin (DCX) [115].Thus, the downregulated expression of neuronal DCX may be mediated by the miRNAs in the exosomes. DCX, as a microtubule-stable protein, is down-regulated in the course of neuronal differentiation. Exosomal-mediated down-regulation of DCX might be vital in the program of the nervous system. During the growth of the nervous system, oligodendrocytes can provide differentiation signals to neurons. The type of DCX can initiate neuronal precursor cell differentiation and also allow mature neurons to be polarized [116]. As a result, the miRNAs carried by exosomes released by oligodendroglial precursor cells may play an essential role in neuroplasticity post SCI.

## 7 Conclusions and future perspectives

Transferring information through circulating vesicles is considered a third way of intercellular association, as crucial as intercellular contact-dependent signals and soluble molecular delivery signals [93,112]. Extracellular vesicles, subdivided into exosomes, microvesicles (MVs), and apoptotic bodies, can transport proteins, lipids, mRNAs and miRNAs. In particular, exosomal miRNA has been shown to regulate protein expression in recipient cells and have functional role *in vivo* [48,117]. It has been reported that treatment by exosomes in the early stage after SCI could attenuate neuronal cell apoptosis [118]. For example, systemic injection of MSCs derived exosomes could promote recovery of SCI rats by increasing the expression level of anti-apoptotic protein B-cell lymphoma 2 (Bcl2) and markedly reducing the activity of pro-apoptotic protein Bcl-2-associated X protein (BaX) [119,120]. In addition, other studies also demonstrated that the anti-apoptotic effect induced by MSC-exosomes targeting Wnt/β-catenin signaling pathway [121] and MSC-exosomes could also reduce neuronal apoptosis by inducing autophagosome formation through improving the expression of autophagy-related proteins, including LC3IIB and Beclin1 [122,123] (Fig. 2).

Moreover, when SCI occurs, the injured spinal column is hypoxic. MSCs derived exosomes, which full of phosphatase and tensin homologous small interfering RNA (ExoPTEN), can significantly enhance the angiogenesis and axon regeneration in the damaged spinal cord by reducing PTEN expression in rats [30,124] (Fig. 2). More importantly, MSCs derived exosomes are also found to decrease the permeability of the blood-spinal- cord barrier (BSCB), enhance its integrity, and promote axon regeneration by down-regulating the NF-κB p65 signaling pathway in pericytes [125]. Furthermore, our previous work also indicated that schwann cell-derived exosomes could repair SCI by attenuating chondroitin sulfate proteoglycans (CSPGs) deposition through activating the Toll-like receptor II 2 on astrocytes [126].

MiRNAs have been shown to bind to mRNA in cells and silence their expression and participate in post-transcriptional regulation. A miRNA can have hundreds of target mRNAs and interact with other miRNAs to regulate post-transcriptional mRNA expression [69]. Previously, miRNAs can only exert their modulatory function within the cell to regulate mRNA expression instead of being secreted outside the cell and act on other cells [70]. The miRNAs cannot exert their regulatory effects if they existing in the extracellular matrix with no protection and no target cells. However, the miRNAs in exosomes can not only been well protected by the phospholipid bilayer and then escape the hydrolysis of RNases, but also receive targeting effect of exosomes, which can act on specific cell populations [71,127,128].

Accumulated researches have indicated that exosomes are capable of mediating cell-to-cell communication [[129], [130], [131]], packaging and transporting miRNAs to new cells and participating in regulating gene expression. Nowadays, exosomal miRNAs are regarded as novel biomarkers for cervical cancer prediction and diagnosis [131,132]. Moreover, BMSCs-derived exosomal miR-150–5p can inhibit the apoptosis of cardiomyocytes and improve the cardiac function via targeting Bax [133,134]. In addition, exosomes derived from BMSCs contained higher miR-29 and miR-24 and lower miR-21, miR-34, and miR-378. These exosomal miRNAs can improve cardiac function by attenuating fibrosis and inflammation in the rat model of myocardial infarction [133,135].Except for MSCs, neurons, microglia and oligodendrocytes, emerging studies also indicate that astrocytes-derived exosomal miR-26a may impact neuronal function and morphology [136] and infiltrated macrophages after SCI could aggravate BSCB integrity breakdown by delivering exosomal miR-155 through activating the NF-κB pathway [137]. Nevertheless, it has not been well studied whether exosomes can accurately transfer miRNAs from their parent cells to their target cells, and the functions of regulating post-transcriptional translation and contribution of SCI and TBI are not transparent.

According to the traditional understanding, most of the carriers for communication between different cells are proteins. However, it has not been found that cells can also transport nucleic acids from one cell to another by encapsulating vesicles and exerting their regulatory effects on target cells [26]. Is this regulation vital in cell's growing, differentiating, as well as function? Does it also participate in various pathophysiological processes that include damage to the CNS and regeneration after SCI? The structure and function of multicellular individuals are inseparable from the information and material exchanges between cells. However, the detailed mechanism of communication between cells is still not well known. As the individual organisms evolve toward higher levels, the communication between cells will also be more accurate, stable, and rapid. The appearance of synapses is a way of precise communication between cells. Exosomal microRNA work on specific cell populations also suggests that they are a more accurate and stable means of the cell-to-cell communication.

## Funding

This work was supported by the following funding: 10.13039/501100001809National Natural Science Foundation of China (Project Number: 81772342, 82072439), Key Program of 10.13039/501100006606Natural Science Foundation of Tianjin (19JCZDJC36300), 10.13039/100011259International Cooperation Program of 10.13039/501100001809National Natural Science Foundation of China (81620108018).

## Consent for publication

Not applicable.

## Availability of supporting data

Not applicable.

## Declaration of competing interest

The authors declare no conflict of interest.
